# Intersections in the digital society: cancel culture, fake news, and contemporary public discourse

**DOI:** 10.3389/fsoc.2024.1376049

**Published:** 2024-03-18

**Authors:** Lucia Picarella

**Affiliations:** Faculty of Law, Universidad Católica de Colombia, Bogotá, Colombia

**Keywords:** cancel culture, digital society, fake news, public discourse, democracy

## Abstract

This article critically examines the intricate relationship between cancel culture and fake news, shedding light on their collective impact on current societies. The changing social landscape, marked by the transition from the “network society” to the “platform society,” has given rise to unprecedented phenomena such as cancel culture. Rooted in social media complaints, cancel culture intersects with the dissemination of intentionally created false information, forming a complex web of dynamics. The study explores the multifaceted nature of cancel culture, its unintended consequences and the nuanced definitions surrounding it. The synthesis of erasure culture and fake news prompts critical reflections on the democratization of information, the protection of fundamental rights, and the potential risks to democracies of an unbridled online narrative. As digital networks continue to play a central role in everyday life, understanding and addressing these challenges is essential to maintaining a balanced discourse that upholds democratic values.

## 1 Introduction

The intrinsic complexity of sociocultural phenomena necessitates an approach that effectively enhances understanding of these phenomena. This need becomes even more apparent in the current historical context, where there is a transition from the “network society” as defined by Castells (1996). This society is characterized not only by the consequences of technological innovation and changes in capitalist structures but also by cultural transformations based on individual freedoms and social autonomy to express identity claims. This transition is toward the “platform society” as described by van Dijck et al. (2018), wherein platforms serve as spaces for the exchange of communicative practices, forms of coexistence, and participation in public life. These platforms also encompass technologies that enable both individuals (individual or collective) and institutions to interact and pursue their goals. In this new ecosystem, novel phenomena emerge, such as digital political subcultures, among them we will pose attention on the “cancel culture.” The latter represents a significant phenomenon within this context, highlighting the complexity of social and cultural dynamics evolving within the platform society. However, there is considerable confusion surrounding the concept of cancel culture, both in terms of its actual meaning—often mistakenly considered synonymous with wokeness and call-out culture, or even politically correct—and its precise development. However, the effects it produces are quite clear. The introduction of this terminology into common language is noted between 2019 and 2020, but the term begins to strengthen as a political claim action since 2015 within Black Twitter—a community of Black Twitter users capable of mobilizing a significant number of people to moderate racist and misogynistic discourse—with hashtags such as #canceled or #“x”isover, where “x” denotes an individual or organization (Roos, 2020). Subsequently, debates on “cancel culture” gained prominence around 2017, driven by the #MeToo movement. This movement, responding to the public revelation of sexual harassment and assault by Harvey Weinstein, combats sexual abuse and violence against women through public denunciation campaigns and boycotts of well-known and powerful figures accused of misconduct. In 2020, the term gained further relevance with the actions of the Black Lives Matter movement, which erupted following protests over the killing of African American George Floyd by the Minneapolis police. The phenomenon of “cancelation” is not new but dates to practices in ancient Greek society and the Roman Empire. In Book III of Politics, Aristotle shows that the polis instituted ostracism as a practice to exile those who were too rich or politically influential from the city for some time, using this practice for sectarian purposes (not for the public good, but as a means to control political power), which they hid behind the justification that the condition of the banned persons was controversial in relation to the principles of equality that were spreading. In the Life of Aristides, Plutarch relates the annual practice of the ostracism vote: citizens would write the name of an undesirable politician and if the person received more than 6,000 votes was banished from Athens for 10 years. The aim of the Roman Empire’s practice of *damnatio memoriae* was to eradicate the “canceled” person from future memory because public figures, statements, and representations are part of social memory, and their function is not mere commemoration: they are also a vital expression of cultural identity. This is why statues, portraits, and public documents promulgated during life were eliminated. The deletion of personalities, inscriptions, philosophical (the death of Socrates is a first example), scientific, or religious theories because they were not aligned with the dominant views can be considered as a precursor to the practices of cancel culture. The phenomenon of the scapegoat to be erased has always existed, but today it is amplified by social networks and digital ecosystems that seem to serve the precise communication strategies of “woke capitalism” (Douthat, 2018). Indeed, reflection on the phenomenon of cancel culture necessitates an observation of the broader dynamics with which this phenomenon is linked. Through a precise communicative and marketing strategy, capitalist structures have co-opted socio-political causes by masking—via washing (greenwashing, purplewashing, queerwashing, etc.)—social accountability and support for progressive and minority struggles (environment, feminism, Afro and LGTB rights, etc.). The apparent woke consciousness (from the slang of African American movements) is used instrumentally to maximize its own profits.

The new battleground is thus the cultural sphere, used to initiate the new woke, washing, or puritan crusades of current fragmented societies.

## 2 A critical examination of the political subculture of cancel culture

But what defines the term cancel culture in practice? In this case, there is no unanimity, although different definitions share common elements. Broadly, it can be defined as “attempts to ostracize someone for violating social norms” (Norris, 2020, p. 2) or more narrowly as “the practice of withdrawing support for (or canceling) public figures and companies after they have done or said something considered objectionable or offensive” (Lizza, 2020). Another perspective sees it as “the withdrawal of any kind of support […] for those who are assessed to have said or done something unacceptable or highly problematic, generally from a social justice perspective” (Ng, 2020, p. 263). Alternatively, “‘canceling’ is an expression of agency, a choice to withdraw one’s attention from someone or something whose values, (in)action, or speech are so offensive, one no longer wishes to grace them with their presence, time, and money” (Clark, 2020, p. 88). As observed, each definition has its nuances, but, as argued by Tandoc et al. (2022), there are common elements synthesized as “(a) the public shaming of unacceptable behavior, and (b) withdrawal of support, which are (c) motivated by wanting to see the target persons experience some form of consequence or penalty due to their actions (e.g., losing employment and other revenue streams) or to ensure these persons are socially banished” (p. 3). What emerges from these common characteristics of diverse definitions? Primarily, the act of “canceling” predominantly occurs through social media denouncements, facilitated by the anonymity provided by some platforms (Tandoc et al., 2022). These denouncements closely resemble public condemnations, aiming to enforce adherence to societal rules. Furthermore, concrete actions range from simple “unfollowing” to more active measures like “boycotting,” which seeks to refrain from purchasing products of a certain brand and persuading others to do the same. In shedding light on the concept of “cancel culture,” it can be unequivocally stated that, on one hand, this practice employs strategies akin to consumer boycott logic to withdraw support from brands and companies, thereby damaging their reputation. On the other hand, it predominantly utilizes social media to “shame” individuals or organizations deemed “guilty”—the so-called phenomenon of “shitstorm” (Sdrigotti, 2018). Its intent is to exert pressure for sanctions ranging from restricting access to public platforms, damaging reputation, and consequently ending careers, to inciting legal actions. It follows that cancel culture is not synonymous with wokeness culture, call-out culture, or political correctness, although they are often confused. As previously mentioned, the term wokeness is derived from the slang of the Afro movements and originally referred to raising awareness and sensitization about the structural and systematic inequalities and violence suffered by this community. Currently, this concept has expanded to include “to any area of oppression and social inequality related to gender, sexuality, violence against women, or any legally unprotected group. Instead of promoting equality, it privileges the weakest social categories; instead of proposing concrete measures for improvement, it fights on the basis of cultural symbols” (Madrid Gil, 2023, p. 24). By contrast, call-out culture is a much more recent concept, based on the power of communication and online platforms with the aim of publicly denouncing, “calling” attention to an issue, or a behavior or statement through digital surveillance and deterrence (Loveluck, 2020). The debate on the concept of politically correct language also has ancient roots, as traces can already be found with the Christian language campaigns to replace pagan denominations that did not correspond to the new religious values of the Roman Empire. Throughout the centuries, political correctness has been imposed by the ruling elites on all spheres (social, cultural, scientific, and religious) and in its modern interpretation the concept goes back to the Marxist-Leninist vision of “alignment” with the ideals of the Communist Party, and then extended in the second half of the 20th century to the civil rights claims of minorities and social movements. According to Hughes (2010), politically correct language refers to a type of language that is more or less consciously used by a social group that considers it in line with its beliefs, values, and ideals. In this sense, therefore, what is not considered “aligned” is censored by the same social group based on terms considered offensive and non-inclusive. These definitions show that there are very blurred boundaries between these concepts and the phenomenon of cancel culture, which at this point we can consider in a broad sense as an exasperation of the three previous dynamics directed at affecting mainly political or star system personalities through the action of cancelation by many people (as opposed to call-out). In fact, the ways of action of cancel culture do not stop at public criticism but oscillate from the removal of any form of support to boycotts, from the repudiation of historical facts/personages or literary and cinematographic pieces to the censorship and cancelation of the subject from public, social, and professional life. The aim of the action of cancel culture does not seem to be so much the restoration of historical and factual truthfulness, but rather the imposition of opinions and ideologies perpetrated through social media activism by the dominant group that establishes what is the dominant opinion. Having clarified that these political subcultures are not synonymous and differ in both the recipients of the practice and the effects they produce, it is evident that while these subcultures may give a voice to marginalized populations for various reasons, they also bring forth debates on significant issues constituting the perverse effects of such practices. On one hand, there are processes of democratization linked to freedom of expression, potentially leading to a reduction in the plurality of opinions. On the other hand, there is the issue of fake news, which significantly distorts phenomena in terms of coverage dedicated to specific related events and uniformity of narrative frames (Mangone, 2022). This accentuates certain themes, concepts, or mental categories while sidelining others. This directs critical reflection on the actual democratization of public and social spaces facilitated by the internet and digital ecosystems, on the protection of the common good and fundamental rights, and on the potential radicalization of a form of “tyranny of the minority” (Goggin, 1984; Bishin, 2009). This, achieved through fake news via cancel culture, nullifies any form of dialectical and deliberative pluralism. Platformization atomizes the public/private separation, and the centrifugal and fluid logic of technological ecosystems transforms the public sphere from a space of communication, confrontation, and social dialogue into a fragmented, contradictory, polarizing space. The concentration of power in the hands of the new digital oligarchies creates a new “post-public sphere” (Sorice, 2000). Through precise communication strategies the new capitalist oligarchies organize public and social space by reducing deliberative pluralism to the point of replacing it with a standardized social discourse coordinated by social networks. This is directly related to the dynamics of the cancel culture: the new narrative imposed as politically correct penetrates all areas and establishes the rules of the game of the new cultural wars conducted with the cancelation of inconvenient and non-aligned content. This creates an obscure “disrupting democracy” (Bertelsmann Foundation, 2018) based on the opposition between woke vs. purity. Since the last century, this conflict has been played in the universities with the purity intervention in the teaching programs directed at safeguarding conservative values, and thus erasing all texts by authors belonging to ethnic and social minorities, or topics relating to race, sexuality, etc., and reciprocally with woke interventions directed at erasing the former. In current societies, there is a dangerous radicalization in both directions. The famous Letter on Justice and Open Debate (2020) highlights the toxicity of this radicalization for debate and democratic pluralism because censorship, cancelation, and ostracism for different opinions, previously the prerogative of conservative right-wing sectors, is spreading in support of woke ideological conformism. In this sense, there is a depoliticization of the claims for social justice and the critical consciousness that accompanied these struggles (e.g., BLM, MeToo). This turns into an extremization of wokeism and of virtual group identity, which becomes a mission of control and cancelation of any opinion not considered correct by a specific community. A mission imposed and played on platforms and social networks. Recall in this perspective Barack Obama’s criticism (BBC, 2019) against the attitude of young people on social networks who confuse activism with the simple overstatement of unconscious judgment through a tweet or a #.

Is it possible to overturn power relations simply by deleting the enemy, nullifying the possibilities of dialogue, and creating new forms of tyranny? In our opinion, the impression that this is intended to provoke by taking advantage of the phenomenon of cancelation and fake news masks the will of the new oligarchies to avoid conscientization on problems (such as inequality, discrimination, lack of equity, and social justice) that are structural and systemic.

## 3 Cancel culture and fake news shape contemporary public discourse

The development of the mass media and, in recent decades, the widespread introduction of information and communication technologies (ICT), multimedia, digitalisation, interactivity and even the improvement of artificial intelligence into everyday life, require an adaptation of the perception and interpretation of reality according to a simultaneous multiplicity of linguistic codes and statistical algorithms. The speed of information demands that the social sciences critically confront the conceptual and argumentative debate between technological determinism and systematization of the notion of the “new” in relation to the dimensions of the socio-cultural sphere. Digital networks are now an integral part of daily life, serving as a means of entertainment, information, and interaction. The widespread dissemination and influential capacity of fake news, however, have allowed for the alteration of thinking patterns and attitudes of the public, further fostering a form of control and acceptance of the “order” that one seeks to establish. Since the Enlightenment, the proliferation of fake news has played a catalytic role on public opinion: Catholic false interpretations of the Lisbon earthquake of 1755 prompted Voltaire to denounce religious domination; fakes published in the United States press during the 1800s about the supposed crimes committed by African-Americans reinforced racist sentiment and provided the basis for the Nazi regime’s anti-Semitic propaganda; the sensationalism of the late 19th century press about the presence of alien civilizations on the moon was used to increase newspaper earnings and circulation (Soll, 2016; Yeoman, 2022). In more recent times, during the COVID-19 pandemic, there was a veritable “infodemic” (Nguyen, 2020) a massive overexposure and dissemination of information and fake news facilitated by the fluidity and homogenization of current informational processes. And still numerous politicians (e.g., Trump, Bolsonaro, and Salvini) have used fake news profusely during their populist campaigns. According to Meneses (2018), it is crucial to distinguish fake news (a term that appeared in the late 19th century) from false news. Although social networks have led to a significant overlap between the two terms, the author argues for the correct use of the term false news to refer to journalistic errors that may result from incompetence, irresponsibility, or superficiality. Fake news, on the other hand, should be understood as intentionally created false and misleading information. In this case, a direct relationship is established between information process, communication, and power. In fact, the misinformation and focus on a packaged block of fake news represent the main strategy to guide public opinion by constructing ideologically and polarized opinion “bubbles.” The fragmentation of public opinion in polarized bubbles is a strategy implemented by the dominant elites to maintain control and to normalize their practices by generating, especially through platforms and social networks, a narcotization and homologation of the user-consumer (as already highlighted by the Frankfurt School’s visions). Through sensationalism, the manipulation of information and fake news, the status quo is established and anything that is not perceived as conforming is canceled. The link between the phenomenon of cancel culture and the massive spread of fake news is evident. According to Zuboff (2019), the main instrument of “surveillance capitalism” is the proliferation of fake news: in line with what we analyzed previously, the pervasive risk of aggression and punishment for exposing one’s ideas, fierce and unarticulated criticism in terms of content, intertwine, and feed a reactionary polarization that manifests itself in the action of canceling.

From the individual to the collective, from the social sphere to the public sphere, the removal of confrontation can be very dangerous for democracy and pluralism. As already mentioned, a critical reflection on the necessary re-democratization of public and social space is urgently needed. Classical theories on the concept of the public sphere were based on the functional separation between what is public and what is private. According to these theories, in a social dimension the notion of the public sphere refers to the integration and participation of citizenship, while in a political dimension it refers to the autonomy of self-legislation, and as the point of union between the two dimensions, communication intervenes, which allows—in the deliberative perspectives of Habermas (1985)—the coordination and understanding between all the subjects and actors involved. The dynamics of action of platformization and social network operate in the public-social sphere through fake news and the cancelation, and through this action the two dimensions expand and blur.

Despite numerous media literacy activities, fact-checking services set up by platforms and newspapers to detect fake news, codes of conduct and ethical and responsible information, and the laws on artificial intelligence that have just been passed by the European Union (AI Act), the interests of oligarchic lobbies, the phenomenon of cancel culture and fake news run at an unstoppable rhythm and seem to be able to circumvent any regulatory obstacles. This makes it imperative to counter these trends to protect democratic freedom and pluralism. If culture is the playing area, then it is precisely from culture and critical education that we must start again. In this sense we agree with Nussbaum’s view on the need to understand the complexity of current societies through autonomous thinking and open dialogue, supplanted by hyper-specialized (neo-liberal) models of education that eliminate “narrative imagination.” All over the world we see examples of praxis of popular critical education, of alternative journalism, of the creation of community media, aimed at conscientizing citizens and opposing the passivity, conformism and ideological massification necessary for the survival of the dominant structures. In this light, for example, we recall the numerous Freire-inspired practices of popular critical pedagogy that have marked an indelible impact and continue to be, in the Latin American context, an important reference point for conscientizing people and fighting for the transformation of highly unequal societies. Similarly, the Italian organization *Parole Ostili* has created an interesting project of civic education paths to promote widespread consciousness against fake news from childhood. With the creation of the Manifesto of Non-Hostile Communication, and by collaborating with schools, universities, the private sector, and public institutions, this organization has initiated free educational courses to accustom students to reading and non-hostile communication not only online but also offline, and to promote a widespread and virtuous consciousness of individual responsibility, respect for oneself and others, and the environment.^1^

The fragmentation of current societies is the fertile ground for the fluid models imposed by woke capitalism, and like a spiral it infects and imposes itself in the same language as alternative sectors, often blocking the results of efforts at change. A new conscious subjectivity and a re-signification of social space must be rearticulated from the educational and cultural base because a return to a critical praxis can provide the basis for framing other possible worlds. In this sense, universities and education in general can get back into the game and restore to education the sociopolitical component that has been progressively eliminated by capitalist models. To really make a difference, it is not enough to train technicians to serve the most powerful, but it is necessary to prepare conscious and critical subjects capable not only of analyzing but also of counteracting current socio-political problems (Titus, 2021). What is needed, then, is a return to an education that is again political practice, enriched through partnership with communities, with social movements, with institutions: an “education for change” (Titus and Potter, 2004).

## 4 Conclusion

The analysis of cancel culture, coupled with the proliferation of fake news, unveils a complex web of dynamics shaping contemporary public discourse. Cancel culture, characterized by public shaming and social media strategies, interlaces with the spread of misinformation, contributing to the challenges presented by the infodemic (Zarocostas, 2020). The interplay between cancel culture, fake news, and their impact on public perception raises critical questions about the democratization of information, the protection of fundamental rights, and the potential risks associated with unchecked online narratives. This interplay highlights the multifaceted nature of cancel culture, extending beyond its immediate targets to influence public narratives and perceptions. The synthesis of cancel culture and fake news underscores the need for a nuanced understanding of their impact on societal dynamics. Cancel culture, operating through social media denouncements, not only shapes public discourse but also amplifies false narratives, contributing to the dissemination of misinformation. The canceling phenomenon, often driven by genuine concerns for justice and accountability, can inadvertently lead to the silencing of diverse perspectives and a potential erosion of democratic values. As fake news becomes intertwined with cancel culture, there is a risk of undermining the democratization of information and fostering a “tyranny of the minority” that suppresses pluralistic dialogue, because if it is a categorical imperative to fight against all forms of discrimination and exclusion, this does not imply “washing” or “cutting” contradictions, errors, and horrors. In contrast, it is necessary to understand and confront different interpretations and narratives because only in this way we can read reality and experiment new alternatives that can block atomization, passivity, and annihilation.

The confluence of cancel culture and fake news necessitates a careful examination of their implications for democratic discourse, information integrity, and the protection of fundamental rights. As digital networks continue to play an integral role in daily life, addressing the challenges posed by cancel culture and misinformation requires a balanced approach that upholds the principles of free expression while safeguarding against the detrimental effects of unchecked narratives. The risk of the absence of this counterbalance is the loss of democracy. Fighting fake news, the dynamics of cancel culture, and the extremes of woke-washing tendencies without censoring pluralism and freedom of thought requires a great joint effort that must start from the sphere of culture and critical education. We need an education in practical politics, which is not an abstract topic, or a form of indoctrination or ideological propaganda (Titus, 2016), but on the contrary can be realized through the construction of new models and strategies that enable the formation, action, and bottom-up organization of a democratic citizenship.

## Author contributions

LP: Writing – original draft.

## Footnotes

^1^ https://paroleostili.it/educazione-civica-a-scuola/
